# What Do Secondary Schools Need to Create Healthier Canteens? The Development of an Implementation Plan

**DOI:** 10.3389/fpubh.2021.683556

**Published:** 2021-06-23

**Authors:** Irma J. Evenhuis, Ellis L. Vyth, Femke van Nassau, Lydian Veldhuis, Marjan J. Westerman, Jacob C. Seidell, Carry M. Renders

**Affiliations:** ^1^Department of Health Sciences, Faculty of Science, Vrije Universiteit Amsterdam, Amsterdam Public Health Research Institute, Amsterdam, Netherlands; ^2^Department of Public and Occupational Health, Amsterdam UMC, Vrije Universiteit Amsterdam, Amsterdam Public Health Research Institute, Amsterdam, Netherlands; ^3^Netherlands Nutrition Centre, The Hague, Netherlands

**Keywords:** behavior change methods, intervention development, food environment, adolecence, implementation plan, school health (MeSH)

## Abstract

**Introduction:** The Netherlands Nutrition Centre developed guidelines to improve the availability and accessibility of healthier food products in Dutch canteens. This paper describes the development of an implementation plan to facilitate implementation of Guidelines for Healthier Canteens in Dutch secondary schools.

**Materials and Methods:** In cooperation with stakeholders (i.e., school/caterer managers/employees, school canteen advisors, researchers) and based on theory, we developed an implementation plan in three steps. First, we identified factors that impede/facilitate stakeholders to create a healthier school canteen during 14 interviews. Second, 25 experts discussed and prioritized these identified factors in an expert meeting. Third, we translated these factors into tools to be included in the implementation plan, by making use of behavior change taxonomies and evidence-based implementation strategies.

**Results:** The plan aims to support stakeholders in implementing healthier school canteens and consists of five tools: (1) tailored advice based on an online questionnaire to assess schools' and stakeholders' context and the Canteen Scan (i.e., an online tool to assess the availability and accessibility of food/drink products); (2) communication materials with information and examples; (3) online community for support by sharing experiences/questions; (4) digital newsletter as reminder/support; (5) fact sheet with students' needs/wishes to tailor the canteen.

**Discussion:** This study illustrates how collaboration between science, policy and practice resulted in a tailored implementation plan aimed to support schools to adhere to school canteen policy. This development serves as a good example for researchers, health promotion policymakers, and practitioners how to create an implementation plan that fits the needs of stakeholders.

## Introduction

Despite the known benefits of healthy dietary behaviors, most people including adolescents do not comply with dietary recommendations (1, 2). It is known that interventions to stimulate a healthy dietary behavior should start at an early age as healthy eating habits developed during childhood and adolescence are likely to persist into adulthood (3, 4). Especially adolescents are known to be prone to adapt unhealthy behaviors, as they are moving on to more autonomy, are developing their own identity, and are developing habits, including dietary ones (3, 4). This makes stimulating a healthy dietary pattern in this age group very important. Healthy school food environments encourage adolescents to make healthier choices (5, 6). In this context, it has been demonstrated that school food policy, among others a healthier school canteen, can stimulate healthy dietary behaviors among youth (7, 8). A school canteen offers food and drinks at school via a tuck shop, a cafeteria, vending machines, or combinations and many students purchase drinks, snacks and meals during their school day at this canteen. In the Netherlands adolescents (aged 9–18) consume 15% of their total food and drink intake per day at school (9). Even though most Dutch students (aged 12–18 year) bring their own lunches from home, they buy complementary foods (snacks and drinks) in the school canteen and around school (10).

Since 2003, the Dutch Healthy School Canteen Program supports secondary schools in creating healthier school canteens (11). The program is coordinated by the Netherlands Nutrition Centre, and financed by the Dutch Ministry of Health, Welfare and Sports. Due to this governmental endorsement the Netherlands Nutrition Center is able to offer free support to all Dutch schools. In the program, school canteen advisors (nutritionists) from the Netherlands Nutrition Centre visit Dutch schools to provide information and advice, send regular newsletters and maintain a website with information and examples about a healthier canteen. This program has been shown to lead to greater attention to healthy nutrition in the school food environment and a small increase of healthier products offered in the cafeteria (11–13). The need to expand and reformulate criteria for healthier canteens emerged as the government increased their focus on healthier canteens (14, 15) and due to practical experiences and further developed scientific insights about for example nudging. The Netherlands Nutrition Centre developed therefore the “Guidelines for Healthier Canteens” in 2014 (16, 17). These guidelines are applicable to school canteens, canteens of sports clubs and worksite cafeterias and include next to availability, also criteria to increase the accessibility of healthier food and drink products (17). This set of guidelines is more extensive than the previous one, and it is unclear to what extent the Healthy School Canteen Program, in its current form, is sufficient to support implementation of these updated guidelines. It is therefore recommended to develop specific implementation support, as practical and feasible implementation support plans can improve the uptake, implementation, maintenance and effectiveness of school canteen policy (18–23).

In the last decade, implementation science has recognised the need for theory as the basis for the development of implementation plans, resulting in several theories, models and frameworks to guide this process (24–26). Although the steps described in these theories differ, it is acknowledged that such developments should combine both scientific evidence and input from practice. Also, it needs to take into account schools' contextual factors, as well as the needs of involved stakeholders, to be able to align the tools to the different needs of practice (27–29). An evidence-based implementation plan therefore consists of a combination of implementation tools, based on evidence-based implementation strategies, affecting factors that hinder implementation according to stakeholders (26, 30, 31). Although studies have shown that tailored implementation strategies can support schools in improving their food environment, for example through education, modelling, training, monitoring and feedback (18, 19, 32, 33), scientific knowledge about which specific strategies are needed to support Dutch schools in implementing the Guidelines for Healthier Canteens is unknown. To enhance reproducibility, allow for comparison with other studies, and to increase use in practice, a full description of the development and content of an implementation plan is necessary (30, 34, 35).

This study illustrates the application of a stepwise systematic method for the development of an implementation plan to support the implementation of the Guidelines for Healthier Canteens, aimed at creating healthier canteens (cafeteria and vending machines) in Dutch secondary schools. The study combined behavior change and implementation theories with input of practice to develop an evidence-based implementation plan.

## Materials and Methods

### The Dutch Guidelines for Healthier Canteens

The implementation plan was developed to support implementation of the “Guidelines for Healthier Canteens” in Dutch secondary schools. These guidelines include criteria on both the availability and accessibility of healthier foods and drinks (including tap water) and an anchoring policy. The guidelines distinguish three incremental health levels: bronze, silver and gold. According to these guidelines, school canteens should offer a majority of healthier products and promote these products through accessibility criteria (17). Healthier products are defined as the foods and drinks included in the Dutch nutritional guidelines the “Wheel of Five,” such as fruits, vegetables, whole grain bread, low fat dairy and water (36), and products that, while not included in the “Wheel of Five,” contain a limited amount of calories, saturated fat and sodium (17). In addition, accessibility is defined by nine criteria to promote these healthier products. These criteria include strategies for product placement (5 items) and product promotion (4 items), such as placement of healthier products at the most eye-catching locations and at the cash-desk, attractive presentation of fruit and vegetables and promotions and discounts are restricted to healthier products (17).

To create healthier school canteens various stakeholders can be involved in different ways. Dutch school canteens can be run by the school itself, by an external catering company, or by a combination of these two. As mentioned, schools can receive support from school canteen advisors from the Netherlands Nutrition Centre and, in some municipalities, local community health promotors also support schools. In most schools, a teacher or facility manager coordinates the involved activities in consultation with the school management. The school canteen itself is mostly run by the canteen manager or canteen employee, of the school itself or an external caterer. Sometimes, students and/or parents are involved in volunteering in the canteen or contribute to the preparation of food.

### Design

**Figure 1:**
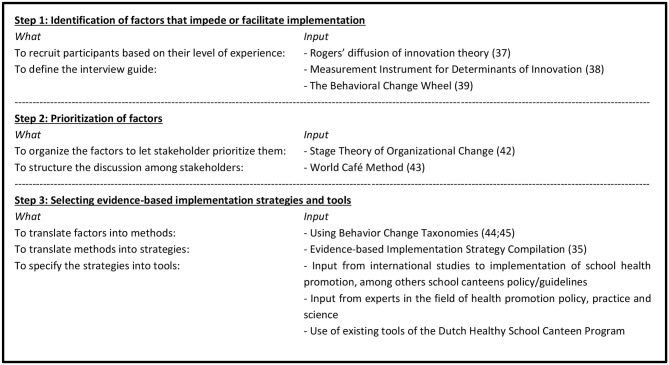
The three-step approach used to develop the implementation plan.

This study, conducted between January and October 2015, involved three steps to develop the implementation plan guided by the “Grol and Wensing Implementation of Change Model” (26) and the Intervention Mapping protocol (31) (see Figure 1). Both models integrate and emphasize the use of theory, evidence and stakeholder involvement and have overlapping steps (26, 31). The Implementation of Change Model was chosen because it provides clear guidance for the need assessments and selection of determinants to change. It consists of six steps from developing a proposal for change to the evaluation and adaptation of the implementation plan. For this study, the three middle steps were applicable: 3) the needs assessment, 4) the selection of implementation strategies, and 5) the development of the implementation plan. For the selection of implementation strategies, the Intervention Mapping approach provided a clear guidance to select behavior change methods, implementation strategies and materials. To summarize, the development of our implementation plan consisted of three steps (see Figure 1). These were: (1) identification of factors that impede or facilitate implementation; (2) prioritization of these factors; and (3) selecting evidence-based implementation strategies and tools.

### Identification of Factors That Impede or Facilitate Implementation

#### Participants

We conducted 14 semi-structured interviews with 18 different stakeholders to identify experienced and expected factors that may impede or facilitate creating a healthier school canteen using the guidelines. Invitations were sent to 15 stakeholders, one of whom was unable to attend due to organizational changes. Four other participants proposed being interviewed together with an involved colleague. We used purposively sampling to recruit participants with a different range of experiences and opinions. We recruited “users” (i.e., people who decide about the product offer and product display and will potentially use the “Guidelines for Healthier Canteens”), and “stakeholders on organization level” (i.e., school and caterer managers). “Users” included school canteen advisors of the Netherlands Nutrition Centre (*n* = 2), school canteen employees (*n* = 1), and school canteen managers (*n* = 5). “Stakeholders on organization level” included school canteen caterers (*n* = 7), school directors (*n* = 2), and a food supplier (*n* = 1). Second, we sampled participants based on their experiences with a healthier canteen, in accordance with Rogers' diffusion of innovation theory (37), as innovators (several years of experience, *n* = 6), majority (some experience, *n* = 10), and laggards (no experience, *n* = 2). By doing this we were able to get a broad insight of expected and experienced needs. This classification of participants was made in agreement with experts at the Netherlands Nutrition Centre, the coordinator of the Healthy School Canteen program, who map the stage of all Dutch schools toward a healthier canteen.

#### Instrumentation and Procedure

Written informed consent was obtained. During the interview, participants received the guidelines which had not yet been disseminated. They were asked to reflect on the guidelines and to indicate what kind of support they would like to receive in order to implement them. The topic list drew upon determinants of The Measurement Instrument of Determinants in Innovations (MIDI) and the Behavioral Change Wheel (BCW) (38, 39), and was optimized on basis of the interviews. The MIDI is a systematically designed tool to measure determinants of innovations that may affect its implementation (38). The BCW is a method for characterizing and designing behavior change interventions, based on the synthesis of 19 behavior change frameworks (39). The main topics were context, experiences, opinions about the guidelines, desired support and solutions and completion. The interviews were performed by a researcher trained in conducting and analyzing qualitative research, with a second researcher taking notes during the interviews. The audio-taped interviews were between 59 and 88 min, and took place between March and May 2015. As the last two interviews did not reveal any new information, it was decided that data-saturation was reached. The interviews were transcribed verbatim, and the summary was validated by each participant.

#### Data Analysis

The thematic content approach was used to analyze the data in three steps: open (label excerpts of the transcripts with descriptive codes), axial (create codes that reflects multiple text fragments and create interpretative codes) and selective coding (compare codes between interviews, to look for correlations) (40, 41). First, the transcripts were read closely and coded independently by two researchers. These descriptive codes were discussed with a third and thereafter with a fourth researcher. During several discussion meetings, the codes were collated into interpretative codes (themes), which were also discussed in the project team. Third, the first three researchers reviewed the themes for coherence and restructured them into more overarching themes. If controversy remained, the other research members were consulted to come to a decision.

### Prioritization of Factors

We prioritized the identified factors through an expert meeting, to reach consensus about the factors that should be addressed by the implementation plan and to generate potential solutions.

#### Participants

Of the 30 experts invited, 5 were not able to attend. Of the 25 experts who did participate, experts worked in research (*n* = 10), in policy (*n* = 4), and in practice (*n* = 11). Attendees included researchers in the field of implementation science and nutrition, school canteen advisors from the Netherlands Nutrition Centre, school facility managers, and representatives of caterers. The expert meeting was led by an external, neutral chair with a scientific background in the field of Nutrition and minutes were taken by a fellow researcher.

#### Instrumentation and Procedure

In preparation for the expert meeting, we organized the identified factors that may impede or facilitate creating a healthier canteen into three stages derived from the Stage Theory of Organizational Change (42) (1) awareness; (2) preparation; and (3) action. This categorization enhanced that the prioritized factors were spread over all stages of implementation. During the expert meeting, for each of these three stages of change consensus was achieved about which factors were most important and modifiable and should be addressed with the implementation plan (26). First, each participant individually ranked all factors in order of importance. In addition, missing factors could be added by each stakeholder. This was put together and discussed plenary to reach consensus about the prioritization of the factors. Thereafter, the structured discussion method World Café (43) was used to reveal and discuss potential actions in subgroups. This method involved that six subgroups consisting of various stakeholders came up with activities to change one of the six highest ranked factors. Subsequently, each subgroup provided their feedback and additions by switching the factors from group to group, before finally presenting their proposed actions.

### Selecting Evidence-Based Implementation Strategies and Tools

#### Procedure and Data Analysis

To create implementation tools that influenced the prioritized factors, we performed three sub-tasks. The identified factors were translated into (1) behavioral change methods and (2) evidence-based implementation strategies, which were then (3) specified into tools (26, 31).

First, the identified factors were translated into behavior change methods, which are methods that can influence determinants of behavior, and behavior of the implementer (31). To select a behavior change method which adequately addresses the identified factors, behavior change taxonomies were used (44, 45). Guided by these taxonomies and in discussion with three researchers, the prioritized factors were linked to behavior change methods. For example, to increase the determinant attitude, the method Elaboration was selected (45). Second, the behavior change methods were linked to corresponding and effective implementation strategies, techniques to enhance the adoption, implementation and sustainability of a program/guideline (34). In our study, we selected strategies as defined by the evidence-based implementation strategy compilation (ERIC). This compilation has been developed to facilitate the selection of effective strategies (35). Third, the chosen strategies were elaborated into implementation tools by defining the mode of delivery, actor, dose, and the target group (34, 46); using the input from the step 2 expert meeting; and reviewing evidence-based implementation strategies and the current tools of the Healthy School Canteen Program (11, 23, 47–49). To select strategies and to specify the tools, one researcher made a proposal, which was reviewed and discussed with two other researchers. The improved proposal was discussed in the project team. During the selection of strategies and tools, the effectiveness and investment for practice were taken into account (e.g., financial, time consumption, alignment with stakeholders' work processes) (26, 48). To ensure that all prioritized factors are part of the implementation plan, a variety of strategies were chosen. We also aimed for inclusion of a mixture of dose (e.g., once, 6-weekly, or if needed), mode of delivery (e.g., real life, paper-based, internet-based or email) and users (e.g., management, coordinator of school, canteen employee) (26, 46, 50). Final decisions about the chosen strategies and tools were made during discussions with the researchers, organizations and stakeholders in the field; the Netherlands Nutrition Centre, the Community Health Service Amsterdam, the nation organization which aims to improve the lifestyle of youth (JOGG) “Young people at a healthy weight,” caterers and schools. All tools were then bundled into the implementation plan.

## Results

### Identification of Factors That Impede or Facilitate Implementation

As Table 1 shows, the interviews resulted in four themes related to creating a healthy school canteen: (1) individual determinants, e.g., lack of knowledge about the canteen guidelines and healthier food options, and insight into the current level in the canteen; (2) commitment of and collaboration with involved stakeholders, both inside and outside the school, including canteen employees, school management, parents, students, caterer and school canteen advisors; (3) school conditions, such as maintaining the initiated policy, keeping the management involved and receiving enough support, financial and time; and (4) environmental conditions, such as the tension between the school canteen and suppliers outside the school.

**Table 1:** Factors and quotes identified during the interviews and prioritized during the expert meeting.

**Theme**	Related factor^*^	**Related quote from the interviews**^**†**^
Individual determinants of involved stakeholders	**Being motivated and enthusiastic to work toward a healthier canteen** ***(quote 1)***. **Having insight into individual/organizational characteristics. Having insight into the level of their canteen (availability and accessibility)** ***(quote 2)***. **Having insight into options how to improve their canteen** ***(quote 3)***. **Having and applying knowledge, to create a healthier canteen (*****quote 4)***. **Having a positive attitude toward a healthier canteen**. Having positive self-efficacy to perform activities with regard to a healthier canteen. Having a coordinator/management of the school who takes the lead in getting a healthier canteen. Being able to create an action plan to create a healthier canteen. Knowing where to get support.	(1): “The enthusiasm of the staff is very important.” (U1)^†^ (2): “For me it is unclear, which product I can/cannot place in our canteen. [..]” (U3) (3): “The canteen is clean and tidy, but doesn't have an attractive presentation to buy food and drinks like a shop. At this moment, I have no idea how to change this.” (U7) (4): “It is difficult [to decide what a healthier/less healthy product is], you hear conflicting stories.” (O7)
Broad commitment of and collaboration with involved stakeholders inside and outside school	**Having/maintaining good collaboration/support with/from students, parents, teachers, management, caterer, canteen employee** ***(quote 5)***. **All stakeholders having a sense of ownership. Developing healthy school (canteen) policy together**. Having/maintaining good collaboration/ support with/from school canteen advisors *(quote 6)*. community health service, caterer, food supplier *(quote 7)*. Having a school canteen working group with different stakeholders *(quote 8)*. Sharing ideas, aims and experiences about a healthier school canteen inside/outside school *(quote 9)*. Having insight into the target group (students).	(5): “If you want to have behavioral change, you need to have a conversation with parents, students and staff from the school to tune it together.” (O4) (6): “I think I have very good contact with them [school canteen advisors] […] I found them very pleasant to work with.” (U5) (7): “[..] full fat yogurt is not really what I want to serve because then I do not comply with the requirements. So, they [suppliers] offer us a low-fat yogurt alternative, they did it for us.” (U5) (8): “It is important that the caterer involves the students. If the caterer creates wonderful things but the students do not like it, it won't not be a success. So, in that respect I think it's good that all three of us [also school] attend.” (U7) (9): “I do not know what students really want. I'm really curious because I think there are opportunities.” (O9)
School conditions	**Maintaining and monitoring the canteen/activities. The management remains involved, supports the initiated policy and acknowledges that the school has a responsibility to their students to offer a healthier canteen** ***(quote 10)***. Having positive finances in a feasible business model *(quote 11)*. Having and perceiving sufficient time, money, employees and facilities to work toward a healthier school canteen *(quote 12)*. Having confidence and good relationship between school and caterer.	(10): “There is no time, no money and no interest. [..]. We spoke to different facility managers who said they have suggested and proposed ideas but it is simply not on the agenda.” (O3) (11): “I notice that there are no revenue targets for a school. Actually, the goal is to break even, the canteen should not cost money. But the caterer has a revenue model because they need to earn a living.” (O3) (12): “Sometimes you lack time, and then you get a “It's okay like this” attitude.” (U3)
Environmental conditions	Collaboration between nearby food providers and school *(quote 13)*. Having broad support in all school activities *(quote 14)*. Providing a canteen that can compete with food provisions outside school.	(13): “I think it is mission impossible if there are supermarkets around the school that sell all sorts of tempting stuff, but you cannot close your school.” (O6) (14): “I also think it depends on location. It depends on whether there are a lot of tempting places in the area or none at all, but a closed square policy would be the best.” (O8)

* *In bold, the factors prioritized highest in the expert meeting*.

† *Participants number: U, User; O, Stakeholder on Organization level*.

### Prioritization of Factors

Factors were prioritized according to the stage of change a school could be in (i.e., awareness, preparation or action). For the awareness stage, experts emphasized the importance that involved stakeholders are motivated, enthusiastic and have a positive attitude toward creating a healthy canteen. Next, consensus was reached that, at the preparation stage, stakeholders need insight into the current canteen/organizational situation, and that the stakeholders in the school need support from students, parents and colleagues. The management needs to facilitate this support. Finally, it was mentioned that, at the action stage, stakeholders need to be able to apply the knowledge to create a healthy, balanced canteen with regard to the offering and accessibility. In addition, they need to be able to create a financial plan, to maintain the intended policy and to collaborate with students, parents and teachers.

For the whole process of creating a healthier school canteen, the experts emphasized that it is important: (1) to create ownership by stakeholders in the school; (2) to make stakeholders responsible for an action; (3) that involved stakeholders receive support from their organization; and (4) to involve multiple stakeholders in one school in the implementation process, including a visible, committed leader and students. To achieve this, they discussed possible activities to inform step 3, such as measuring the proportion healthier/less healthy products available and accessible in the canteen (according to the guidelines), providing tailored advice, providing examples of healthy canteens and healthier products, and enabling schools to share their experiences to learn from each other's successes and challenges. These options were taken into account in step 3.

### Selecting Evidence-Based Implementation Strategies and Tools

**Figure 2:**
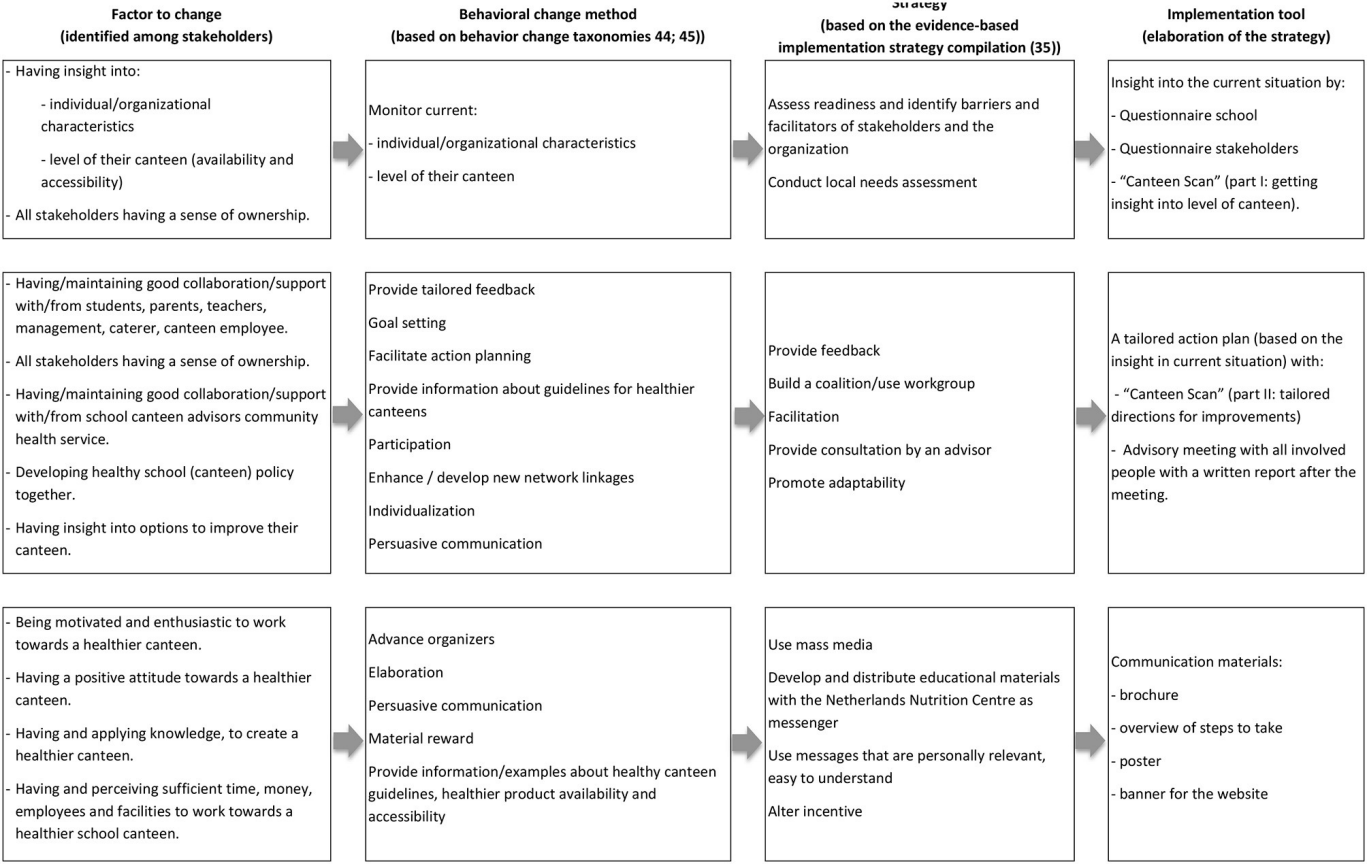
Overview of the translation from factors to implementation tools, via behavioral change methods and strategies.

Describing the prioritized factors as objectives, we translated them into behavioral change methods, implementation strategies and finally specified them into implementation tools (Figure 2). These steps led to multiple implementation tools, both adapted existing and new developed tools. These tools (see Table 2) comprised a questionnaire for the schools and stakeholders as well as the online “Canteen Scan.” The results of these two tools are used as input for the advisory meeting and report. Other tools included communication materials (brochure, poster), newsletters, and a fact sheet with students' needs. In addition, an online community was provided. Advisors of the Netherlands Nutrition Centre were advised to offer all implementation tools to all schools. The tools could be tailored to the different stage of change of the school, the context of the school, the needs of the stakeholders. In particular by the content of the advisory meeting, which was guided by the results of the questionnaires and the Canteen Scan and accompanying tailored actions were formulated together.

**Table 2:** Description of the implementation plan to implement the Guidelines for Healthier Canteens^*^.

**Implementation tool**	**Action and targets**	**Target group**	**Period**
1. Insight into the current situation
*1.1: Questionnaire, school*	An online questionnaire to assess the characteristics of the school. The school specific results provide input for the advisory meeting (38, 51).	Coordinator of the school	At the start, before the advisory meeting
*1.2: Questionnaire, stakeholders*	An online questionnaire to assess stakeholders' characteristics, and their individual and environmental determinants. The school specific results provide input for the advisory meeting (38, 51).	All involved stakeholders	At the start, before the advisory meeting
*1.3: ‘Canteen Scan'*	An online tool to assess the level of the canteen. It provides (I) insight into, and (II) directions for improvement of, the availability and accessibility of food and drink products of the school canteen (52).	Performed by a school canteen advisor of the Netherlands Nutrition Centre. Results and feedback are provided to all involved stakeholders.	At the start, before the advisory meeting
	To create ownership and insight into the changes so far, the school receives information to fill out the Canteen Scan by themselves if they wanted.	Performed by the school coordinator.	After three months
*1.4: Advisory meeting and report*^†^	In one advisory meeting per school, all involved stakeholders are advised about how to improve the canteen by a school canteen advisor of the Netherlands Nutrition Centre. Based on the points of attention, identified with the two questionnaires and the Canteen Scan a concrete action plan will be developed during the meeting. This action plan is created together it will increase ownership and collaboration. After the meeting, a written report based on this meeting is distributed by email.	All involved stakeholders	At the start of implementation
2. Communication materials^†^	Several materials are handed to each school: A brochure about the Guidelines for Healthier Canteens; an overview of the steps to take; a personalized poster; a banner for the schools' website. The materials aim to create motivation and to increase and apply knowledge.	Coordinator of the school, who is asked to share this with other stakeholders.	At the start and halfway implementation
3. Online community	A closed Facebook community for stakeholders, to share their experiences, ask questions and support each other.	All stakeholders	Continuous
4. Digital newsletter^†^	A regularly newsletter sent by email. It consists of information and good examples regarding the healthy school canteen. It aims to support, remind and motivate stakeholders.	All stakeholders	Every 6-week.
5. Students' fact sheet	A summary of each schools' own students' wishes and needs with regard to a healthier school canteen, based on the results of a student's questionnaire. It gives schools insight into the opinions of their students and how their students want to be involved.	Coordinator of the school, who is asked to share this with other stakeholders.	Once, 2–4 weeks after the start.

* *This table is adapted from the version published in the design paper (53)*.

† *This tool was an existing tool of the Healthy School Canteen Program, but was improved/adapted to support implementation of the Guidelines for Healthier Canteens*.

## Discussion

In this study we systematically developed a plan to facilitate implementation of the Guidelines for Healthier Canteens in Dutch secondary schools. We integrated the involvement of stakeholders and school canteen advisors, the use of behavior change taxonomies, evidence-based implementation strategies and experiences with the Dutch Healthy School Canteen Program. This resulted in a plan consisting of several tools, supported by practice and evidence, and aligned to the needs of schools. In order to optimize the effectiveness and usability of the implementation plan, the tools cover a range of different doses, modes of delivery and target groups (26, 46, 50).

The implementation plan is designed to address multiple factors which enable or impede implementation of the Guidelines for Healthier Canteens. These factors were identified by different stakeholders. Identification of the needs of stakeholders in implementing school canteen guidelines is an important first step in developing implementation tools (31). In addition, it aims to create a positive environment, which is likely to improve the uptake of the developed implementation plan (50). Our study identified the following factors that can impede or facilitate implementation of healthier canteen guidelines: (1) individual determinants (e.g., positive motivation, attitude toward a healthier canteen); (2) commitment of and collaboration with involved stakeholders; (3) school conditions (e.g., support of management, monitoring the canteen); and (4) environmental conditions (e.g., collaboration with nearby food suppliers). Although our study focused on the implementation of school canteen guidelines, some of the identified factors also enabled health promotion in schools in general, for example good collaboration and clear communication between stakeholders inside school, and support of management (54–56). Supporting ownership is a common and important factor that may facilitate the implementation of school health policy (19, 57). Stakeholders in our study also identified ownership as a need to create a healthier canteen. Such ownership can be increased by creating goals and actions aligned to and in participation with stakeholders and receiving tailored feedback (31, 58). Consequently, in our plan it is advised to invite all stakeholders to the advisory meeting, in order to create aims and actions together. These aims and actions are based on the insights into their characteristics, the school's context and the level of the canteen as obtained through the Canteen scan and the questionnaires.

Our final implementation plan was based on implementation strategies that have been shown to change behavior and thus supporting implementation, such as audit, feedback, monitoring, education, information, incentives and sharing knowledge and experiences (18, 19). McIsaac et al. (19) also emphasized the importance of tailoring tools to the individual needs of schools to support implementation, as it is easier for schools to implement and maintain actions aligned to their system, organizational culture and circumstances (19, 50, 55). That is why the tools have been developed in such a way that they can be tailored to the needs of a specific school and its stakeholders. Whether our implementation tools actually support implementation needs to be further investigated in an already planned effect and process evaluation (53).

A strength of our study is that we developed implementation tools that can be tailored to the needs of a specific school, to the school's context and to the implementation phase, as some schools are just starting with implementing a healthy school canteen while others have been involved in the healthy school canteen for years. One example of an implementation tool that can be tailored is the advisory meeting. This meeting aims to align the actions to the school by discussing common aims, actions and actors for implementation with the involved stakeholders, such as school managers, caterers, school canteen employees and involved teachers.

Another strength of our study is the use of existing theoretical frameworks to guide the development of the implementation plan. Moreover, we have integrated scientific knowledge in the field of implementation with practical insights within every step. For example, we used the existing categorization of determinants from the “Measurement Instrument of Determinants in Innovations” (MIDI) and the “Behavior Change Wheel” (BCW) to develop the topic list for the interviews. This allowed us to identify impeding and promoting factors at both individual and organizational level, as well as on innovation and the broader contextual level. We expect that this continuing alignment between practice and scientific knowledge will assure a sustainable implementation.

A third strength of our study is the detailed description of the development of our implementation plan. Such a comprehensive description enables comparison of results between studies, and gaining further knowledge about selection of implementation strategies (29, 34, 35, 59). A clear description of the development and content of the implementation tools can also increase its use in practice (29). A review of effective strategies to improve implementation of school-based health programs recommends performing high quality studies to improve the evidence of effective implementation of school canteen policy (33). This study contributes to this area of knowledge.

Although it is widely recommended and has proven to facilitate sustainable implementation, collaboration with practice during the development of an implementation plan is not always applied (30, 48, 50, 55). Therefore, another strength of our study is the intensive collaboration with stakeholders with a diverse background in research, policy and practice throughout each step of our development process (30, 31). This wide range of stakeholders revealed a great diversity of factors that varied across schools' characteristics and stage of change. For example, the input of practice was given by school canteen managers and schools' management, but also by school canteen advisors and school caterers. These advisors and caterers are involved in multiple schools, and have therefore a broad insight into the factors facilitating or hindering a healthier school canteen and the needs of different schools. This comprehensive insight increases the change that an implementation plan is usable and feasible for a wide range of schools and stakeholders (50).

## Limitations

One limitation of our study is that we did not involve students as stakeholders during the development of our implementation plan. Since involvement of students in creating a healthier canteen was identified as a need in our study, and also in previous research (55), and valuing their input is found to be important (57, 60), we advise schools to take into account students' opinions and needs in the process of creating a healthier canteen. We facilitate this by offering each school the student fact sheet, which contains their students' needs and wishes. In addition, during the advisory meeting, schools are encouraged to involve students, although how to do this is not specified to allow for local tailoring. While this freedom for schools to choose how they want to involve students can be regarded as a strength, as schools can align this to their own cultural and organizational habits, it could also be a limitation, as schools are not supported in this process.

Another possible limitation is that our implementation plan does not consider the outside school environment, such as supermarkets and cafeterias, which may encourage students to consume unhealthy foods and drinks during or around school time. As interviews with stakeholders identified concerns about this outside school environment, in the advisory meeting we encourage schools and school canteen caterers to address this topic. One example of a solution was to create school policy to oblige students to stay in the school yard during breaks.

Another identified point of concern, and possible limitation was the influence of parents, who have a major influence on and are also responsible for their children's nutritional behavior (61). Good collaboration with and involvement of parents is therefore important. Although our implementation plan advises schools to involve parents, they indicate that they perceive this as difficult. Future studies should investigate how parents can be reached and how they can be involved in creating a healthier canteen (55, 62).

## Conclusions

This study illustrates the application of a stepwise systematic method for the development of an implementation plan. This resulted in an evidence-based implementation plan, that allows tailoring, aimed to support secondary schools in creating a healthier canteen. Further studies to investigate the effects of this implementation plan in practice are planned. Although this plan needs to be adjusted for use in other contexts, this study can be used as an example approach to develop an implementation plan that is supported by both science and practice.

## Data Availability Statement

The raw data supporting the conclusions of this article will be made available by the authors upon request, without undue reservation.

## Ethics Statement

Ethical review and approval was not required for the study on human participants in accordance with the local legislation and institutional requirements. The patients/participants provided their written informed consent to participate in this study.

## Author Contributions

CR, EV, and JS wrote the project application. IE was the executive researcher of the study, supported by CR, EV, MW, LV, and FN. IE drafted and all the other authors helped to refine the article. All authors approved the final version.

## Conflict of Interest

The authors declare that the research was conducted in the absence of any commercial or financial relationships that could be construed as a potential conflict of interest.
